# Single-cell time-lapse imaging of intracellular O_2_ in response to metabolic inhibition and mitochondrial cytochrome-*c* release

**DOI:** 10.1038/cddis.2017.247

**Published:** 2017-06-01

**Authors:** Heiko Düssmann, Sergio Perez-Alvarez, Ujval Anilkumar, Dmitri B Papkovsky, Jochen HM Prehn

**Affiliations:** 1 Department of Physiology and Medical Physics, Royal College of Surgeons in Ireland, 123 St. Stephen’s Green, Dublin 2, Ireland; 2 Centre for Systems Medicine, Royal College of Surgeons in Ireland, 123 St. Stephen’s Green, Dublin 2, Ireland; 3 Luxcel Biosciences, Western Gateway Building, Cork, Ireland; 4 School of Biochemistry and Cell Biology, University College Cork, Cork, Ireland

## Abstract

The detection of intracellular molecular oxygen (O_2_) levels is important for understanding cell physiology, cell death, and drug effects, and has recently been improved with the development of oxygen-sensitive probes that are compatible with live cell time-lapse microscopy. We here provide a protocol for the use of the nanoparticle probe MitoImage-MM2 to monitor intracellular oxygen levels by confocal microscopy under baseline conditions, in response to mitochondrial toxins, and following mitochondrial cytochrome-*c* release. We demonstrate that the MitoImage-MM2 probe, which embeds Pt(II)-5,10,15,20-tetrakis-(2,3,4,5,6–pentafluorophenyl)-porphyrin as oxygen sensor and poly(9,9-dioctylfluorene) as an O_2_-independent component, enables quantitative, ratiometric time-lapse imaging of intracellular O_2_. Multiplexing with tetra-methyl-rhodamine-methyl ester in HeLa cervical cancer cells showed significant increases in intracellular O_2_ accompanied by strong mitochondrial depolarization when respiratory chain complexes III or IV were inhibited by Antimycin A or sodium azide, respectively, and when cells were maintained at ‘physiological’ tissue O_2_ levels (5% O_2_). Multiplexing also allowed us to monitor intracellular O_2_ during the apoptotic signaling process of mitochondrial outer membrane permeabilization in HeLa expressing cytochrome-*c*-eGFP, and demonstrated that mitochondria post cytochrome-*c* release are able to retain their capacity to respire at physiological O_2_ despite a decrease in mitochondrial membrane potential.

Aerobic organisms require a constant supply of molecular oxygen (O_2_) to produce ATP through oxidative phosphorylation by mitochondria, a process that also leads to the formation of reactive oxygen species (ROS).^1^ The response to O_2_ levels in mammalian tissues is tightly regulated by specific genes and signaling pathways in order to maintain cell bioenergetics and survival.^2^ Severe fluctuations in O_2_ levels may lead to anoxia (no oxygen), hypoxia (decreased availability of O_2_) or hyperoxia (increased O_2_ levels), each condition capable of inducing cell and tissue damage. Because of uncontrolled cell proliferation, cancer cells are often exposed to tissue hypoxia. Many cancer cells are therefore specifically equipped to adapt and survive hypoxic periods.^3, 4^

Similar to hypoxic conditions, mitochondrial cytochrome-*c* (cyt-*c*) release during apoptosis also induces a bioenergetics crisis, as cyt-*c* shuttles electrons between complexes III and IV.^5, 6, 7^ Many cancer cells are resistant to caspase activation,^8^ and when caspase activation is compromised, cancer cells may survive the bioenergetics crisis induced by cyt-*c* release, as the fraction of cyt-*c* remaining in the intermembrane space after equilibration with the cytosolic compartment may still be able to contribute to respiratory chain activity.^9, 10, 11^ This enables mitochondria to sustain intracellular ATP in the absence of further mitochondrial degradation. This process is facilitated through enhanced extracellular glucose uptake, another key bioenergetics alteration of cancer cells.^9^

Because of the key role played by the mitochondrial respiratory chain in the control of cell survival during apoptosis, O_2_ sensing represents an important method for the study of cancer energy metabolism and bioenergetics responses to metabolic inhibitors or mitochondrial cyt-*c* release.^12^ Therefore, the development of new O_2_ sensing and imaging protocols that enable measurements of oxygen levels in single living cells and during asynchronous, apoptotic cell death relative to other physiological parameters is of great interest to the cell death and bioenergetics community.

Significant progress has been made in the field of molecular O_2_ detection by optical sensing.^13^ The advantages of this technique are its sensitivity, accuracy and non-invasive nature.^14^ Quenching of phosphorescence has become an important method for measuring O_2_ by optical sensing.^15^ Phosphorescence quenching relies on the ability of O_2_ to quench the emission of excited triplet state molecules. In biological systems, phosphorescence quenching is highly specific to O_2_, since oxygen is the only small molecule dynamic quencher present in sufficiently high concentrations.^16^ Advantages of phosphorescent probes include high specificity, fast response, high sensitivity, stable calibration and various readout parameters such as intensity and lifetime. However, most of the probes developed still could not satisfy all the requirements for O_2_ measurement in high-resolution imaging modalities in long-term experiments because of lack of compatibility with other probes, requirement of special imaging hardware, limited uptake into cells, or significant phototoxicity.^17^ Detection techniques such as the Whalen-style platinum electrode^18^ allow for the measurement of O_2_ consumption at the single cell level,^19^ but only deliver data for one cell at a time. Other optical intracellular oxygen sensing probes and techniques including Clark-type oxygen chips^20, 21^ often require highly specialized equipment such as fluorescence life time microscopy technologies.^21, 22^ As many laboratories routinely use confocal or epifluorescence time-lapse imaging, there is a significant need for the development of probes for these applications.^23, 24^

In this study, we evaluated the utility of a nanoparticle-based phosphorescent probe, MitoImage-MM2, consisting of the O_2_-sensitive phosphorescent reporter dye (PtTFPP) and the O_2_-insensitive component (PFO) embedded in a cationic polymer,^25^ for confocal time lapse imaging. We demonstrate that MM2 senses changes in oxygen concentration at single cell level in response to hypoxia, metabolic inhibitors and mitochondrial cytochrome-*c* release, and demonstrate its suitability for multiplexing with additional single cell fluorescent probes. Importantly, we also demonstrate that cell culture environment needs to be adjusted to more physiological conditions,^26^ to more reliably detect alterations in cellular O_2_ levels and mitochondrial bioenergetics.

## Results

### MM2 accurately measures O_2_ concentration

**Figure 1:**
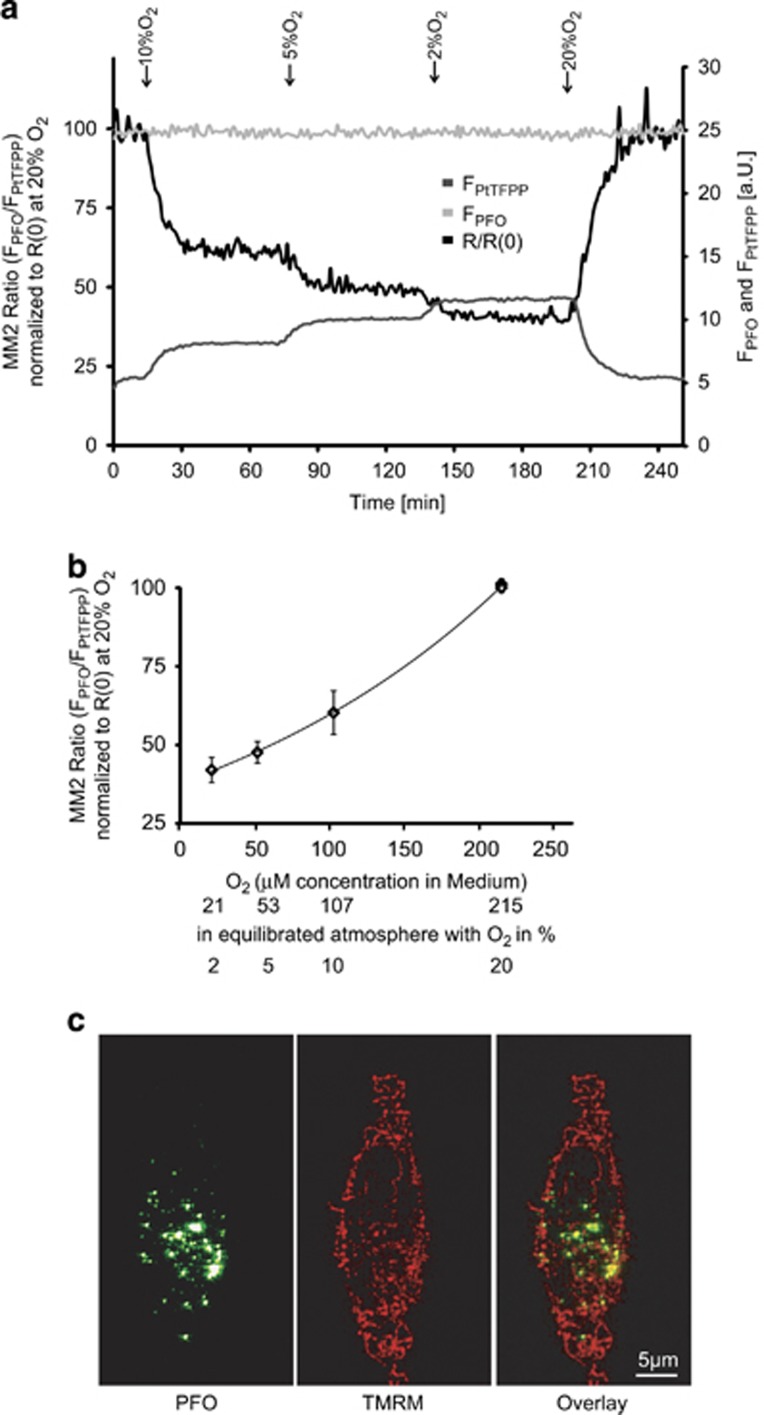
Performance of MM2 in ratiometric intensity-based oxygen sensing. (**a**) MM2 (10 *μ*g/ml) was added to Willco dishes and covered with 1 ml mineral oil. Representative field of view-traces of fluorescence intensity ratio *R*=*F*_PFO_/*F*_PtTFPP_ normalized to *R*_0_ for MM2 probe exposed to different oxygen concentrations (20, 10, 5 and 2% O_2_). (**b**) Calibration curve resulting from measurements as in (**a**) for the MM2 fluorescence intensity ratio at indicated oxygen concentrations (215, 107, 53 and 21 *μ*M O_2_). Data are shown as ±S.E.M. *p<0.05 indicates difference between 225.74 *μ*M O_2_ at time point 0 min against different oxygen concentrations (215, 107, 53 and 21 *μ*M O_2_). Experiments were repeated three times on separate preparations with similar results. (**c**) Representative images showing uptake of the MM2 probe and mitochondrial TMRM in a HeLa cell. MM2 nanoparticles inside the cell, shown with one 1.2 *μ*m thick optical section (FWHM, Scale bar, 5 *μ*m)

The MM2 probe has been previously shown to be sensitive to O_2_ and compatible with specialized detection platforms such as FLIM systems and time-resolved fluorescence (TR-F) readers.^25, 27^ Here we evaluated the capability of the ratiometric MM2 probe to monitor changes in O_2_ concentration in HeLa cells loaded with MM2 nanoparticles, using a confocal live cell imaging setup. First, we explored the response of the probe alone under different O_2_ concentrations. To this end we measured the MM2 ratio signal *R*=(*F*_PFO_/*F*_PtTFPP_) using 10 *μ*g/ml of the probe in glass bottom cell culture dishes at varying O_2_ concentrations on the live cell imaging setup, using the stage incubator described in Materials and Methods. In order to account for the variability of *R*(*t*=0 min) between experiments, we normalized *R* to its start value *R*_0_=*R*(20% O_2_). The on-stage calibration indicated a strong, linear correlation between the normalized MM2 intensity ratio *R*/*R*_0_ and the oxygen concentration equilibrated to 20, 10, 5 and 2% O_2_ (Figures 1a and b). The dependence of the PtTFPP intensity on the O_2_ concentration is clearly visible in Figure 1a, while the PFO intensity remained stable during the calibration with 2, 5, 10 and 20% oxygen in the stage incubator atmosphere. Fitting to an exponential function provided the calibration equation O_2_ [*μ*M]=1/0.0045 exp (*R*/*R*_0_/38.01), with an *r*^2^=0.9997, representing an optimal fit. To confirm that the un-quenching of PtTFPP and therefore the drop in *R*/*R*_0_ was reversible, O_2_ in the buffer was again equilibrated to ambient O_2_ at the end of each calibration experiment (Figures 1a and b).

### MM2 is effectively loaded into HeLa cells

We next conducted a series of experiments to establish the best uptake conditions in HeLa cells. We incubated HeLa cells with 10 *μ*g/ml MM2 probe in medium containing 10, 5, 2 and 1% of fetal bovine serum (FBS) for 16 h. This was required as the concentration of heat inactivated serum in the culture medium has been shown to affect the intracellular concentration of many probes and nanoparticles.^28^ We identified 1% FBS as the optimum condition to deliver the probe into the cells without affecting cell viability (data not shown). To investigate the distribution of the MM2 nanoparticles in cell culture, we obtained stacks of confocal images with an optical slice thickness of 1 *μ*m of live HeLa cells incubated with the MM2 probe and 30 nM of the cationic dye TMRM, an indicator of mitochondrial membrane potential, (ΔΨ_M_). Confocal analysis indicated that the MM2 signal derived from intracellular vesicles located in proximity to mitochondria (Figure 1c).

### Effect of mitochondrial chain inhibitors on intracellular O_2_ levels of HeLa cells maintained at ‘supra-physiological’ (20%) or ‘physiological’ (5%) ambient O_2_

**Figure 2:**
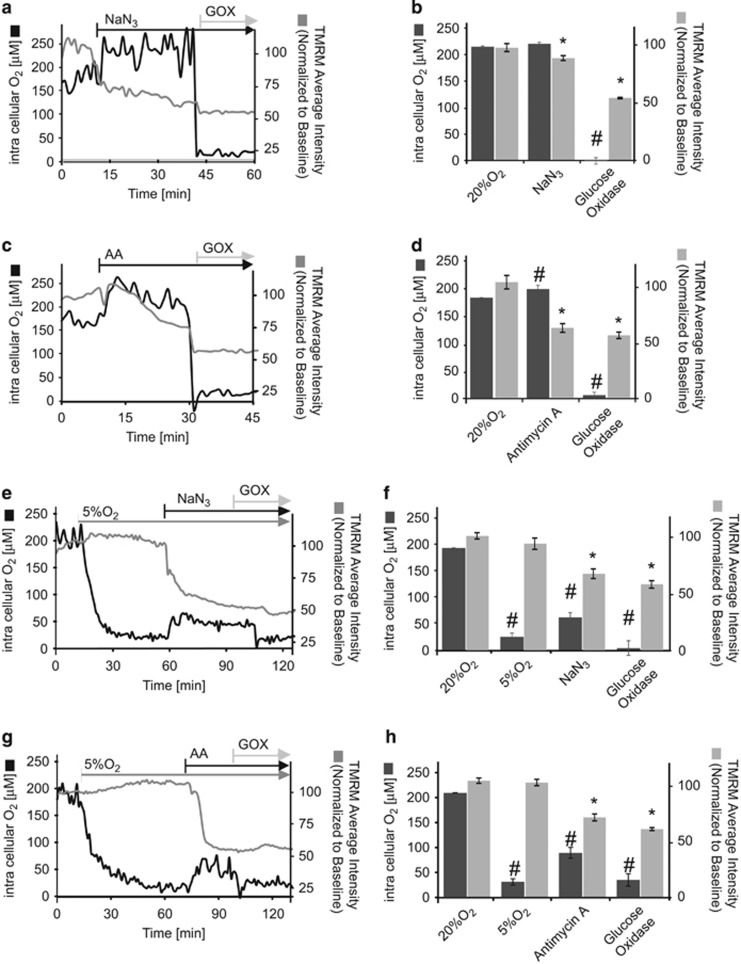
MM2 probe senses oxygen consumption by HeLa cells at 20 and 5% O_2_. (**a**,**b**) Kinetics of MM2 probe intensity ratio in response to different electron transport chain inhibitors at the time points indicated at 20% O_2_. HeLa cells were loaded with MM2 probe (10 *μ*g/ml) and TMRM (30 nM) and mounted on a confocal microscope stage. Cells maintained at 20% O_2_ and in medium without glucose supplemented with 10% FBS and 2 mM sodium pyruvate were exposed to Antimycin A (AA, 10 *μ*M) (**a**) or NaN_3_ (0.2 mg\ml) (**c**). Experiments were finished by addition of glucose oxidase (100 *μ*g/ml). (**b**,**d)** Quantification of MM2 fluorescence intensity ratio at 20% O_2_ and during the (**c**) AA or (**d**) NaN_3_treatment. *and # indicate a significant difference with *p*<0.05, paired samples *t*-test, experiments were repeated two times on separate preparations. (**e**,**g)** Representative traces of changes in MM2 fluorescence intensity ratio following (**e**) AA (10 *μ*M) or (**g**) NaN_3_ (0.2 mg\ml) addition in cells maintained at 5% O_2_. Cells incubated in medium without glucose supplemented with 10% FBS and 2 mM sodium pyruvate were maintained at 5% O_2_ for 60 min to allow equilibration and then (**e**) AA or (**g**) NaN_3_ was added to cells. Experiments were ended by addition of glucose oxidase (100 *μ*g/ml). (**f**,**h)** Quantification of MM2 fluorescence intensity ratio at 5% O_2_ and during (**f**) AA or (**h**) NaN_3_. Data are shown as ±S.E.M. *, ^#^*p*<0.05, paired samples *t*-test indicates a significant difference between 5% O_2_ in at 45 min against AA or NaN_3_ treatment at 60 min, experiments were repeated three times on separate preparations

We next explored the effect of mitochondrial electron transport chain (ETC) inhibitors on intracellular oxygen levels and ΔΨ_M_ as detected with TMRM. It has been shown that HeLa cells in a glucose-deprived medium shift their energy metabolism predominantly towards oxidative phosphorylation.^29^ All experiments were therefore carried out with HeLa cells in glucose-free medium supplemented with 2 mM sodium pyruvate. HeLa cells loaded with MM2 probe and incubated with 30 nM TMRM were treated with ETC inhibitors, Antimycin A (AA; 10 *μ*M), or NaN_3_ (0.2 mg/ml). AA and NaN_3_ inhibit complexes III and IV, respectively. As expected, inhibition of complex IV (Figures 2a and b) or complex III (Figures 2c and d) resulted in a significant decrease in the TMRM signal. Mitochondrial respiratory chain inhibition should lead to increased intracellular O_2_ levels as oxygen consumption is strongly inhibited under these conditions. However only complex III inhibition induced a significant, detectable increase in intracellular O_2_ when cells were maintained at 20% extracellular O_2_ (corresponding to an extracellular O_2_ concentration of 215 *μ*M). The change of consumption after complex IV inhibition in medium equilibrated in 20% O_2_ allowed us to determine the absolute intra-cellular concentration of O_2_ in HeLa cells at the start of this experiment, assuming *R*=*R*(20% O_2_) in cells treated with Antimycin A. Next we conducted a similar experiment, but reduced extracellular O_2_ from 20 to 5% prior to complex inhibition to monitor responses at a more ‘physiological’ extracellular O_2_ level occurring in normal tissues.^26^ After 60 min of equilibration at 5% O_2_, cells were exposed to AA (Figures 2g and h) or NaN_3_ (Figures 2e and f). Reducing extracellular O_2_ from 20 to 5% resulted in no significant decrease in TMRM average intensity, indicating that ΔΨ_M_ was not compromised by this change. Under these conditions, addition of AA or NaN_3_ induced a significant increase in intracellular O_2_ concentration in response to ETC inhibition that was accompanied by a more pronounced drop in TMRM fluorescence intensity than in ambient O_2_ (Figures 2e and f). Glucose oxidase and glucose was added at the end of the experiment to deplete all O_2_ available in the medium, serving as an internal control for probe specificity to sense O_2_.^27^ Fercher and co-workers previously also showed that the respiratory capacity of cultured cells can be examined with mild stimulation of respiration by addition of low doses of a protonophore imaged with PtTFPP impregnated nanoparticles and phosphorescence life time imaging.^27^

### Hypoxia limits consumption of O_2_ in respiring HeLa cells with only a minor effect on ΔΨ_M_

**Figure 3:**
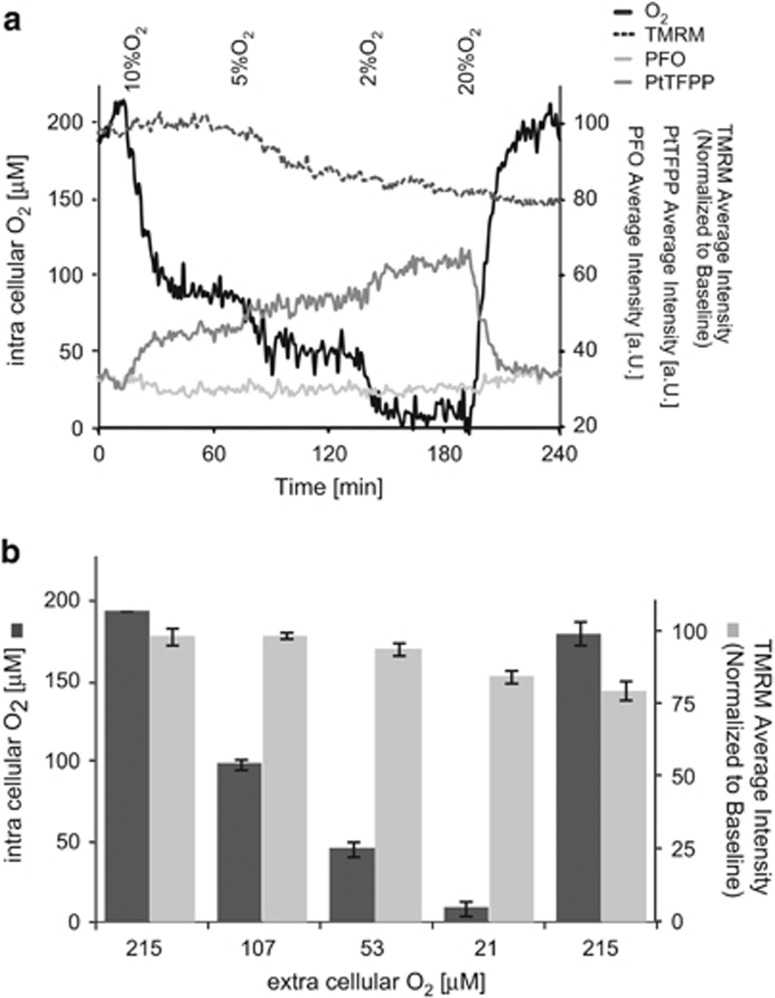
MM2 ratiometric intensity-based intracellular oxygen sensing within HeLa cells. (**a**) Representative kinetics of intracellular oxygen (black) and TMRM (black, punctate) average intensity one HeLa cell in the field of view in medium exposed to different oxygen concentrations equilibrating the medium to 20, 10, 5, 2 and back to 20% O_2_. Single cell kinetics of PFO (light grey) and PtTFPP (dark grey) depict the stability of the PFO and the oxygen sensitivity of PtTFPP respectively. (**b)** Quantification of O_2_ using the MM2 fluorescence intensity ratio at indicated extracellular oxygen concentrations. Data are shown as ±S.D. of three independent experiments in HeLa

Next we analyzed the kinetics of ΔΨ_M_ and O_2_ consumption of respiring HeLa cells during incremental decreases of extracellular O_2_. The O_2_ concentration in the stage incubator was successively reduced from 20 to 10%, 5 and 2% O_2_ which resulted in 215, 107, 53 and 21 *μ*M dissolved O_2_. O_2_ levels were then returned to 20%. Again, we carried out the experiments in glucose-free medium supplemented with 2 mM sodium pyruvate, thereby forcing cells to use the ETC to fuel energy metabolism by consumption of O_2_. The cells were stained with MM2 and TMRM. Interestingly, the change in the extracellular O_2_ concentration from 5 to 2% still resulted in a significant decrease of available O_2_ in HeLa cells without depleting intracellular oxygen completely (Figures 3a and b). ΔΨ_M_ detected with the TMRM probe dropped stepwise with O_2_ being reduced to 5% and then 2%, to significantly lower levels of 95.7±2.0% (S.E.M.) and 85.7±2.2% (S.E.M.) of the initial values. Interestingly, TMRM fluorescence intensity did not recover following the experiment and stayed at 80.9±3.6% (S.E.M.) of the initial value, while O_2_ returned to baseline levels (Figures 3a and b).

### MitoImage-MM2 probe reveals respiration in single HeLa cells which underwent MOMP

**Figure 4:**
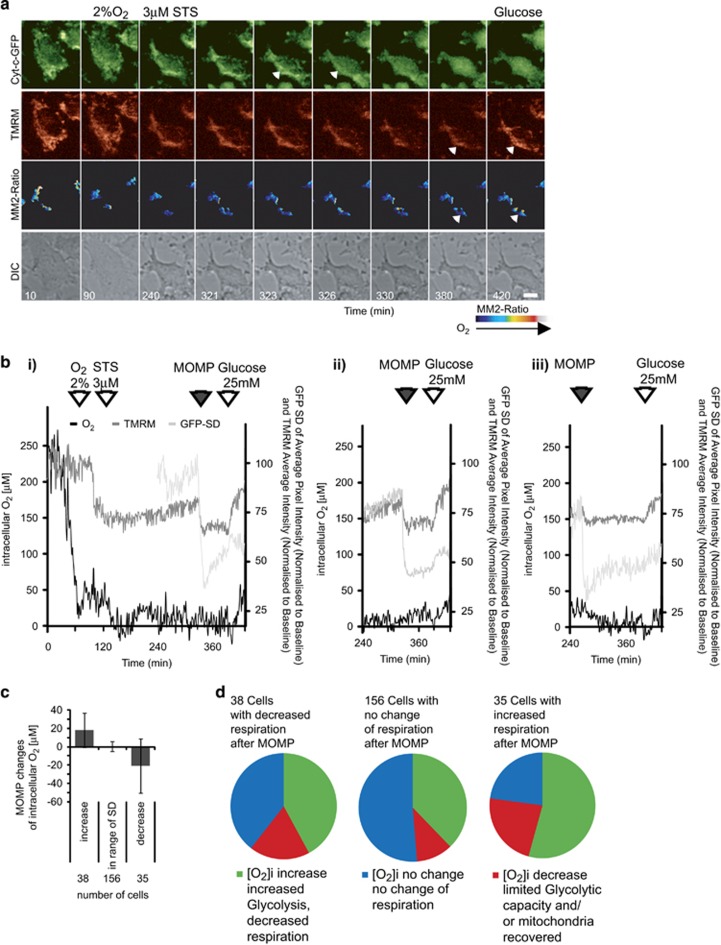
Multimodal imaging of MM2, cyt-*c*-GFP and TMRM at single cell level. Representative single-cell microscopic imaging of HeLa cells expressing cyt-*c*-GFP, loaded with MM2 (10 *μ*g/ml) for 16 h, incubated with 30 nM TMRM, and caspase inhibitor zVAD-fmk (100 *μ*M) 1 h prior to treatment with 3 *μ*M staurosporine (STS, as indicated). The cells were kept in RPMI without glucose, supplemented with 10% FBS, and 2 mM sodium pyruvate. On stage the O_2_ was maintained at 2% for 60 min before treating with STS. (**a)** Representative images of a single cell showing a decrease in TMRM after O_2_ was downregulated from 20 to 2%, cyt-*c*-GFP release as an indicator for MOMP and subsequent further loss of TMRM, followed by recovery of TMRM after addition of 25 mM glucose to the cells (scale bar: 10 μm). The oxygen increase after addition of glucose is visible in the single (**b)** cell kinetics of that cell. The single kinetics of O_2_ (solid black line), TMRM (solid dark grey) and SD of GFP average intensity (solid light grey, only displayed for significant time frame) show that the addition of glucose changes the intracellular oxygen level in the cell which underwent MOMP. The cell on the left (i) did not change respiration after MOMP as indicated by no change in intracellular O_2_ while the cell represented in the middle (ii) showed an increase in intracellular O_2_ indicative of a decrease in respiration. The cell represented by the kinetics on the right (iii) showed a decrease in intracellular O_2_ indicative of an increase in respiration after MOMP. (**c)** Changes in intracellular oxygen concentration in cells undergoing MOMP. Out of 288 cells from 4 experiments, 255 cells showed a release of cyt-*c*-GFP (MOMP, indicated by grey arrowhead in **b**) after addition of STS and 228 before addition of glucose. Of those cells 38 showed increased oxygen concentration, 35 showed a decrease in and 156 showed no change in intracellular oxygen (data shown +/− S.D.). (**d**) Shows the three groups as separated in **c**. Glucose addition caused a heterogeneous response in respiration. A large fraction of all cells responded with a decrease in respiration. The single cell history with increased respiration after MOMP drove the majority towards a decrease of respiration after glucose addition. Cells which had no response or a decrease in respiration after MOMP were less likely to decrease respiration after glucose

We next explored whether multiplexing with the MM2 probe would also allow for simultaneous time lapse imaging of both cyt-*c*-eGFP redistribution and ΔΨ_M_ using TMRM during apoptosis. Previous studies have suggested that oxidative phosphorylation activity and mitochondrial ATP production is still abundant after outer mitochondrial membrane permeabilization (MOMP) when caspase activation is compromised and glucose is available.^9, 11, 30^ HeLa cells over expressing cyt-*c*-eGFP were pre-treated with 100 *μ*M zVAD-fmk and then equilibrated at 2% O_2_ for 60 min to mimic borderline normoxic/hypoxic conditions in tumors. Cells were then treated with the apoptosis-inducing broad-spectrum kinase inhibitor STS to induce the release of cyt-*c*-GFP. We hypothesized that a compromised ETC turnover after cyt-*c* release would cause an increase in intracellular O_2_. Figure 4a depicts a representative image series, which demonstrates the successful multiplexing of MM2 with GFP and TMRM. Single cell cyt-*c*-GFP traces showed a significant decrease in the standard deviation of the pixel intensity of GFP fluorescence, indicating the onset of cyt-*c* release (Figure 4a, arrowheads in GFP channel indicate mitochondria before [323′] and after [326′] cyt-*c*-eGFP release; MOMP is indicated in Figure 4bi by filled arrowheads). The depolarization of ΔΨ_M_ coincided with cyt-*c*-GFP release as reported previously,^9, 31^ but intracellular O_2_ did not increase after MOMP in this cell, indicating that mitochondria were still consuming O_2_. Once MOMP had occurred, we explored the effect of glucose addition on intracellular O_2_ and ΔΨ_M_. The addition of glucose recovered ΔΨ_M_ in this cell (Figure 4a, TMRM; white arrowheads, before [380′] and after [420′] glucose addition). Of note, the addition of glucose also led to an increase in intracellular O_2_ concentration in this cell, suggesting not only a decrease in F_o_F_1_-ATP synthase activity (indicated by increased TMRM fluorescence intensity), but also a concomitant decrease in ETC activity (Figure 4a, MM2, white arrow heads, before [380′] and after [420′] glucose addition). Hence MM2 can be used efficiently in multi-modal setup to measure O_2_ concentrations in living cells without interference with GFP or green excitation/red emission probes such as TMRM.

By analyzing the single cell traces of *n*=228 cells that underwent MOMP during these experiments; we however also noted a significant heterogeneity in responses. MOMP resulted in an increase in respiration in 38 cells (decrease of intracellular O_2_; represented by the single cell kinetics in Figure 4biii), in no change in 156 cells (as depicted in Figure 4bi), or a decrease in respiration in 35 cells (increase of intracellular O_2_; represented by single cell kinetics in Figure 4bii). A quantification of these three types of responses is provided in Figure 4c. In order to investigate whether this was also determining the outcome of respiration changes after glucose addition, we separately analyzed the three response groups (Figure 4d). Interestingly, the majority of cells with a single-cell history of increased respiration after MOMP demonstrated decreased respiration after glucose addition. In contrast, cells which showed no response or a decrease in respiration after MOMP were less likely to decrease respiration after glucose addition. Furthermore, 149 of the 228 cells studied showed a recovery of ΔΨ_M_ (as indicated by an increase of TMRM average intensity) after the addition of 25 mM glucose.

## Discussion

In this report, we evaluated the potential of MitoImage-MM2 to probe alterations in intracellular O_2_ levels and exploited the multiplexing capability of MM2. We found that exposure of HeLa cells loaded with MM2 to different oxygen levels accurately detected intracellular O_2_ concentrations similar to *ex vivo* experiments. To explore whether MM2 reported alterations in O_2_ levels as a consequence of changing mitochondrial respiration, we exposed HeLa cells to Antimycin A and NaN_3_ and detected alterations in O_2_ levels in particular when cells were maintained at more physiological tissue O_2_ levels (2–5%). The complete O_2_ consumption inhibition with NaN_3_ enabled us to quantify the cellular O_2_ concentration in cells kept in standard culture conditions. This then allowed us to calibrate single cell O_2_ kinetics, and to simultaneously measure ΔΨ_M_ kinetics.

MM2 is a second-generation probe for O_2_ imaging, characterized by its PFO and PtTFPP, suitable for confocal and two-photon imaging.^25^ In contrast to the loading of cells with nanoparticles incorporating PtTFPP alone,^27^ MM2 can be used for ratiometric measurements. The dye combination used in MM2 also circumvents detector sensitivity issues with an emission wavelength above maximum at 650 nm,^32^ making it suitable for confocal time lapse imaging.^33^ MM2 was photo-stable with little bleaching and could be calibrated to estimate actual oxygen levels within cells. MM2 is compatible with fluorescent proteins, FITC- and rhodamine-based optical probes. Applications of second-generation phosphorescent probes for O_2_ sensing adds speed,^19, 34^ and probes can be used for plate reader-based assays in parallel.^35^ Interestingly, a cell-impermeable analog of MM2, PtP-C343, modified with polyethylene glycol residues, has recently also been shown to measure O_2_ in brain microvessels when administered intravenously.^22^

Our confocal microscopy experiments indicated that MM2 was taken up and internalized in HeLa cells. While we have not yet defined the intracellular structures that incorporate MM2, a previous study using similar nanoparticles has identified uptake into endosomes in proximity to mitochondria.^27, 36^ Using the settings described in our study, we did not detect toxicity of the probe for up to 24 h after a 16-h incubation period, or phototoxic effects triggered by the image acquisition settings in control time lapse experiments. The detection principle used (quenching of the phosphorescence signal by O_2_) will potentially generate ROS. To avoid this, acquisition settings have to be tested and adjusted to the highest sensitivity and lowest excitation light intensity and exposure time. This comes at the cost of increased noise, limiting the detection of subtle changes of intracellular O_2_. However as we demonstrate in this report, MM2 is a highly interesting probe for detection of the kinetics of intracellular O_2_ concentration by light microscopy, and enables the detection and classification of response types of intracellular O_2_ changes even at low atmospheric oxygen in multiplexing approaches at the single cell level as demonstrated in Figure 4.

Previous findings have shown that atmospheric O_2_, cell density, respiration rate and its dynamics are the major factors influencing the oxygen-sensitive signaling pathways.^37^ We show that alterations in oxygen levels in transformed HeLa cancer cells could be adequately detected at an oxygen level of 53 and even 215 *μ*M (5% O_2_ or 20% in the atmosphere with 5% CO_2_). This represents O_2_ levels higher than found in the human body, which ranges between 9 and 21 *μ*M in healthy and 2.5and 13 *μ*M in cancer tissue,^38^ but still represents an extracellular O_2_ level much lower than atmospheric oxygen (215 *μ*M). At atmospheric O_2_ levels, mitochondrial respiration inhibition with sodium azide did not induce significant alterations in intracellular oxygen levels. This may be due to the fact that dissolved O_2_ at a high concentration (20%) can diffuse and equilibrate rapidly into the cells, rendering it difficult to detect changes in ETC O_2_ consumption. Importantly, as oxygen consumption is central to ATP production via the respiratory chain, this implies that any analysis of cellular bioenergetics, such as mitochondrial membrane potential, ATP levels, ROS production, or NADH/NADPH levels should be performed under controlled oxygen levels.

Because mitochondria are the primary consumer of molecular O_2_ and also control adaptive responses to hypoxia,^39, 40, 41^ cellular O_2_ levels can be an indicator of O_2_-dependent metabolic activities, such as aerobic respiration or oxygen-dependent synthesis and degradation of cellular components,^33, 42^ and acts as a potential site for O_2_ sensing in the cell.^43, 44^ Indeed, we found that simultaneously detected TMRM uptake to be different at 20 and 2% O_2_ when cells fully rely on ETC to maintain their energy metabolism and fully depend on mitochondrial ATP. However, findings from isolated mitochondria suggest that mitochondrial O_2_ availability does not become critically low and ATP production through ETC remains stable until the oxygen concentration falls near anoxic conditions (<0.3%).^45, 46^ Tumor cells harbor the ‘Warburg effect’ which describes a switch from mitochondrial respiration to glycolysis in the presence of oxygen. However, recent studies have highlighted that this ‘switch’ is not complete and tumor cells are well capable of using mitochondria for aerobic ATP production.^47, 48^ Indeed, we show that inhibition of respiratory chain activity with Antimycin A or NaN_3_ significantly increased intracellular O_2_ in cells kept under physiological O_2_ concentration.

Cytochrome-*c* is the main enzyme involved in transporting electrons and binds to oxygen in the ETC.^49^ This, in turn, polarizes mitochondrial membrane potential, which drives proton motive force for ATP synthase to produce ATP.^50^ In our experiments, cyt-*c* release during apoptosis depolarized ΔΨ_M_ but had little effect on intracellular O_2_ levels in the majority of cells, confirming previous reports that released cyt-*c* is still accessible for mitochondrial respiration.^11^ Our study also demonstrates that, when glucose became available in cells which underwent MOMP, most cells responded with an increase in intracellular O_2_, indicating that cancer cells are able to flexibly adapt to extracellular glucose increases post-MOMP with an increased glycolytic activity. Of note, our data also show a significant heterogeneity of bioenergetics responses post cyt-*c* release and glucose addition. This heterogeneity was not unexpected, as previous single cell imaging and mathematical modelling studies from our laboratory indicated a strong heterogeneity in mitochondrial respiration post MOMP, with cells operating different modes of complex V/ATP synthase activity in a given population of cancer cells, depending on the amount of respiration-accessible cyt-*c* and the degree of glycolytic ATP production.^9^ This may also explain our findings that cells with increased respiration after MOMP (excessive respiration accessible cyt-c) were more likely to respond to an increase in extracellular glucose with a decrease in respiration, while cells with limited respiration (and limited accessible cyt-*c*) showed no decrease in respiration as a response to glucose addition.

The direct link between increased glycolytic activity and oxygen consumption in cancer cells that underwent MOMP may also be important in the context of ROS production and HIF-dependent and -independent hypoxia signaling, as both are strongly influenced by intracellular O_2_ consumption and may control demise or recovery of cells post-MOMP.^51, 52^ The findings reported here may therefore also have important implications for tumor cell survival and resistance to therapy.

## Materials and methods

### Materials

RPMI medium without glucose was from Gibco (Biosciences, Dublin, Ireland). RPMI medium, Antimycin A (AA), sodium azide (NaN_3_), glucose oxidase from *Aspergillus niger* and carbonyl cyanide 4-(trifluoromethoxy) phenylhydrazone(FCCP) were purchased from Sigma Aldrich (Tallaght, Dublin, Ireland). Tetramethylrhodamine MethylEster (TMRM), fetal calf serum and Minimal Essential Medium were from Invitrogen (Biosciences, Dublin, Ireland). Staurosporine (STS) was from Axxora (Alpha technologies, Blessington, Ireland). Z-Val-Ala-Asp(O-methyl)-fluoromethylketon (zVAD-fmk) was obtained from Bachem (Heidelberg, Germany). The MitoImage-MM2 probe was provided by Luxcel Biosciences (Cork, Ireland).

### Cell culture

HeLa cells and HeLa cells stably expressing cytochrome-*c*-eGFP (cyt-*c*-GFP,^31, 53, 54^) were grown in RPMI supplemented with 10% FBS at 37 °C in a humidified atmosphere at 5% CO_2_. For the experiments, cells were seeded on glass-bottom culture dishes (WillCo Wells B.V., Amsterdam, The Netherlands), at a density of 20 000 per dish and maintained at 37 °C in a humidified atmosphere of 5%CO_2_/95% air. Experiments were carried out in glucose-free RPMI, supplemented with 10% FBS and 2 mM sodium pyruvate.

### Monitoring changes in O_2_ concentration using the ratiometricMM2 probe

HeLa cells were loaded with 10 *μ*g/ml of MM2 (or co-loaded with TMRM for 30 min in the dark) in RPMI medium supplemented with 1% FBS medium for 16 h at 37 °C. MM2 probe intensity ratio was recorded at different oxygen concentrations (20, 10, 5 and 2% O_2_) in a Willco dish mounted on LSM 5 live Duoscan confocal microscope (Carl Zeiss, Jena, Germany) equipped with a × 40, 1.3 NA Plan-Neofluar oil-immersion objective and a thermostatically regulated chamber (Pecon, Erbach, Germany) at 37 °C in a humidified atmosphere of 5% CO_2_/95% air. CO_2_and O_2_ levels (%) in the stage incubator were regulated using the CTI controller 3700 Digital in combination with an O_2_ controller (Pecon, Erbach, Germany). This unit requires N_2_ to displace O_2_ from the incubation atmosphere. The MM2 probe was excited using 2% of the 30 mW 405 nm DPSS Laser, and the emission was collected through a 415–480 nm band pass and a 570 nm long pass filter using a 565 nm secondary dichroic to split the emission between the two detectors of the LSM 5 live. TMRM was excited at 561 nm, and the emission was collected through a 570–640 nm band pass filter. In HeLa cells expressing cyt-*c*-GFP, GFP was excited with a 489 nm DPSS laser and the emission was detected using a 495–555 nm band pass filter. All images were processed using ImageJ (version 1.45, Wayne Rasband, NIH, Bethesda, MD, USA) and MetaMorph Software version 7.5 (Molecular Devices, Wokingham, Berkshire, UK). The intensity ratio images between the PFO and the PtTFPP (*F*_PFO_/*F*_PtTFPP_) were calculated for all areas of the image with PFO and PtTFPP fluorescence above background noise and after background subtraction. Mitochondrial membrane potential (ΔΨ_m_) changes were measured using the average pixel intensity of TMRM per cell.

### Sodium azide (NaN_3_) and AntimycinA (AA) treatment at physiological (5%) and hyperoxic (20%) O_2_

HeLa cells cultured on Willco dishes and incubated with the MM2 probe (10 *μ*g/ml) for 16 h were co-loaded with TMRM (30 nM) at 37 °C in the dark for 30 min. The Willco dishes with cells were mounted on the stage of an LSM 5 live confocal microscope (Zeiss, Jena, Germany) equipped with a × 40, 1.3 NA Plan-Neofluar oil-immersion objective and a thermostatically regulated chamber (Pecon, Erbach, Germany) at 37 °C in a humidified atmosphere of 5% CO_2_/95% air. Cells were maintained at 20% O_2_ for 10 min to allow for equilibration and then reduced to 5% O_2_ using the equipment described above. After 60 min of equilibration the cells were exposed to NaN_3_ (0.2 mg/ml) or AA (10 *μ*M). TMRM and MM2 probe fluorescence intensities were recorded. Experiments were terminated by addition of D-(+)-Glucose (100 mM) and glucose oxidase (100 *μ*g/ml) to completely deplete the available oxygen. In another set of experiments, Hela cells were maintained at 20% O_2_. After a 10-min equilibration period, cells were exposed to sodium azide (NaN_3_, 0.2 mg/ml) or AA (10 *μ*M) and TMRM and MM2 probe intensity ratio was recorded as described above.

### Induction of apoptosis and simultaneous measurement of oxygen consumption and GFP redistribution

HeLa cyt-*c*-GFP cells were cultured in glass bottom dishes and incubated with 10 *μ*g/ml MM2 probe for 16 h followed by TMRM (30 nM) and zVAD-fmk (100 *μ*M) for 30 min to inhibit caspase activation. After treatment with STS, caspase activation would otherwise trigger mitochondrial demise following MOMP due to cleavage of complex I.^9, 11, 30^ Cells were mounted on stage as described above and maintained in 20% O_2_ for the required 10 min of equilibration. Then O_2_ was reduced to 2% using the equipment described above. After 60 min cells were treated with 3 *μ*M STS. During the subsequent time lapse imaging experiments, glucose was added to the medium as indicated. All images were processed using ImageJ (1.45) and MetaMorph Software version 7.5 as described above. The distribution of cyt-*c*-GFP was calculated using the S.D. of the average pixel intensity as described previously.^31^ A decrease in the S.D. value indicates the redistribution of GFP from punctate (mitochondrial) to homogeneous (cytoplasm and nucleus) patterns. For further analysis of cell responses, cells were grouped into three classes based on their intracellular O_2_ kinetics following MOMP: (a) Cells with an increase in intracellular O_2_ following MOMP (the average of the 10 upstream values+S.D. was smaller than the average of the 10 downstream values following MOMP). (b) Cells with a decrease in intracellular O_2_ following MOMP (the average of the 10 upstream values+S.D. was larger than the average of the 10 downstream values following MOMP). (c) Cells with no change in intracellular O_2_ following MOMP (remainder of cells).

### Statistics

Data are given as means±S.E.M. For statistical comparison, one-way analysis of variance, paired samples *t*-test, Wilcoxon test and Friedman’s test were carried out using SPSS software (SPSS version 23, IBM). Where the *p-*value was<0.05, groups were considered to be significantly different. All experiments were carried out in triplicate unless indicated otherwise.
